# Integrative genomic and immune landscape analysis of intimal sarcomas for emerging therapeutic targets and immunotherapy strategies

**DOI:** 10.3389/fimmu.2026.1723978

**Published:** 2026-03-03

**Authors:** Livia Gozzellino, Alice Costa, Margherita Nannini, Maria C. Nigro, Carmine Pizzi, Francesco Angeli, Luca Bergamaschi, Chiara Baldovini, Barbara Corti, Luisa Di Sciascio, Davide Pacini, Gianluca Folesani, Mauro Gargiulo, Luigi Lovato, Ilenia Motta, Gianandrea Pasquinelli, Annalisa Astolfi, Maria A. Pantaleo

**Affiliations:** 1 Department of Medical and Surgical Sciences (DIMEC), University of Bologna, Bologna, Italy; 2 IRCCS Azienda Ospedaliero-Universitaria di Bologna, Bologna, Italy; 3 Medical Oncology, IRCCS Azienda Ospedaliero-Universitaria di Bologna, Bologna, Italy; 4 Cardiovascular Division, Morgagni-Pierantoni University Hospital, Forlì, Italy; 5 Pathology Unit, IRCCS Azienda Ospedaliero-Universitaria di Bologna, Bologna, Italy; 6 Cardiac Surgery Unit, IRCCS Azienda Ospedaliero-Universitaria di Bologna, Bologna, Italy; 7 Vascular Surgery Unit, IRCCS, University Hospital Policlinico S.Orsola, Bologna, Italy; 8 Pediatric and Adult Cardiothoracic and Vascular, Oncohematologic and Emergency Radiology Unit, IRCCS Azienda Ospedaliero-Universitaria di Bologna, Bologna, Italy; 9 Division of Pathology, IRCCS Azienda Ospedaliero-Universitaria di Bologna, Bologna, Italy

**Keywords:** bioinformatics, checkpoint inhibitors, immune infiltrate, intimal sarcoma, PD-L1

## Abstract

**Introduction:**

Intimal sarcomas are aggressive mesenchymal tumors arising from the tunica intima of large vessels, mainly the pulmonary artery. They are usually associated with *MDM2* amplification. Due to their rarity and scarce sensitivity to chemotherapy, they are characterized by late diagnosis and high mortality. Thus, there is an urgent need to unravel novel therapeutic biomarkers. This study explored the role of the immune infiltrate and molecular profile in an intimal sarcoma cohort to evaluate their amenability to immunotherapy and detect potential targets, apart from *MDM2*.

**Methods:**

Whole transcriptome and whole exome sequencing were performed on 5 intimal sarcoma cases (FFPE) followed by computational analyses, including immune cell profiling, differential gene expression, variant calling and copy number alteration detection.

**Results:**

All samples presented the amplification of *MDM2*, confirming their diagnosis, and the co-amplification of *CPM* and *SLC35E3*. Interestingly, they also showed PD-L1 expression along with a prevalence of CD4+ memory resting T-cells, M2 macrophages and different concentrations of naïve B-cells, CD8+ T-cells and monocytes. The upregulation of immunoglobulins and pathways involved in the immune response (e.g. IL6/JAK/STAT3 and TNF-α via NF-kB signaling, interferon gamma response) further suggested a potential sensitivity to immunotherapy.

**Discussion:**

Our findings provided basic evidence for immunotherapy efficacy in intimal sarcomas and identified potential molecular targets. Further studies involving larger case series are required to validate these results.

## 1 Introduction

Intimal sarcomas (IS) are rare malignant entities of mesenchymal origin arising from the tunica intima of large vessels, mainly the pulmonary artery. They usually affect middle-aged adults causing intraluminal growth with consequent obstruction and possible emboli in near vessels (1–3). IS show an undifferentiated pattern including spindle, epithelioid and pleomorphic cells, also found in undifferentiated pleomorphic sarcomas (UPS) and myxofibrosarcomas. The main biomarker considered useful for IS diagnosis is the overexpression of *MDM2*, the mouse double minute 2 homolog (12q15) (1, 4–6). This event is often associated with amplifications involving 12q12-q15 (*CDK4, GLI1*), 7p11.2 (*EGFR*), 4q12 (*PDGFRA*, *KIT*) and the 9p21.3 (*CDKN2A*) loss (6). Interestingly, *MDM2* drives p53 degradation by a negative feedback loop, which allows cells to bypass cell cycle arrest or apoptosis (7, 8). Consequently, this interaction leads to uncontrolled cell division and growth.

Regarding treatment, localized IS can benefit from surgery sometimes combined with radiotherapy (5). However, since this histotype is extremely rare and symptoms can vary and mimic thromboembolic disease, late diagnosis often occurs leading to tumor expansion and, eventually, metastasis onset (9). Additionally, except for anthracycline-based regimens, intimal sarcomas show scarce sensitivity to chemotherapy (e.g. doxorubicin) and, therefore, poor prognosis with an overall survival rate of 5–18 months (3, 5). Conversely, radiotherapy seems to effectively control tumor growth, enhancing surgery success and diminishing the risk of recurrence (3). Few studies have discussed the role of immune checkpoint inhibitors: the response to pembrolizumab was evaluated in three IS cases, while an enriched tumor microenvironment (TME) was described in seven samples (10, 11). Overall, these findings emphasize the need to uncover alternative therapies and to shed light on the role of the immune infiltrate in the treatment of intimal sarcomas.

Herein, we aimed to investigate the molecular signature and the TME of our cohort by next generation sequencing (NGS) to identify potential targets and any signal of amenability to immunotherapy.

## 2 Materials and methods

### 2.1 Cohort of pulmonary artery intimal sarcomas

The study comprised five cases of pulmonary artery intimal sarcoma whose features are shown in **Table 1**. Their mean age was 50.2 years old and they all presented metastases at the time of the diagnosis, leading to poor prognosis. The molecular analysis was conducted in accordance with the Declaration of Helsinki and was approved by the Institutional Ethics Committee of Policlinico Sant’Orsola-Malpighi, Bologna, Italy (approval number: 95/2013/U/Tess; date: 8 October 2013).

**Table 1:** Classification of our subset of patients (n=5) diagnosed with intimal sarcoma.

ID	Gender	Age	Site	Disease status^a^	Last follow-up
L328	M	50	pulmonary artery	advanced	DOD^b^
L329	M	37	pulmonary artery	advanced	DOD^b^
L330	M	50	pulmonary artery	advanced	DOD^b^
L331	F	45	pulmonary artery	advanced	DOD^b^
L332	F	69	pulmonary artery	advanced	DOD^b^

^a^Disease status at diagnosis; ^b^DOD, Died Of Disease.

**Figure 1:**
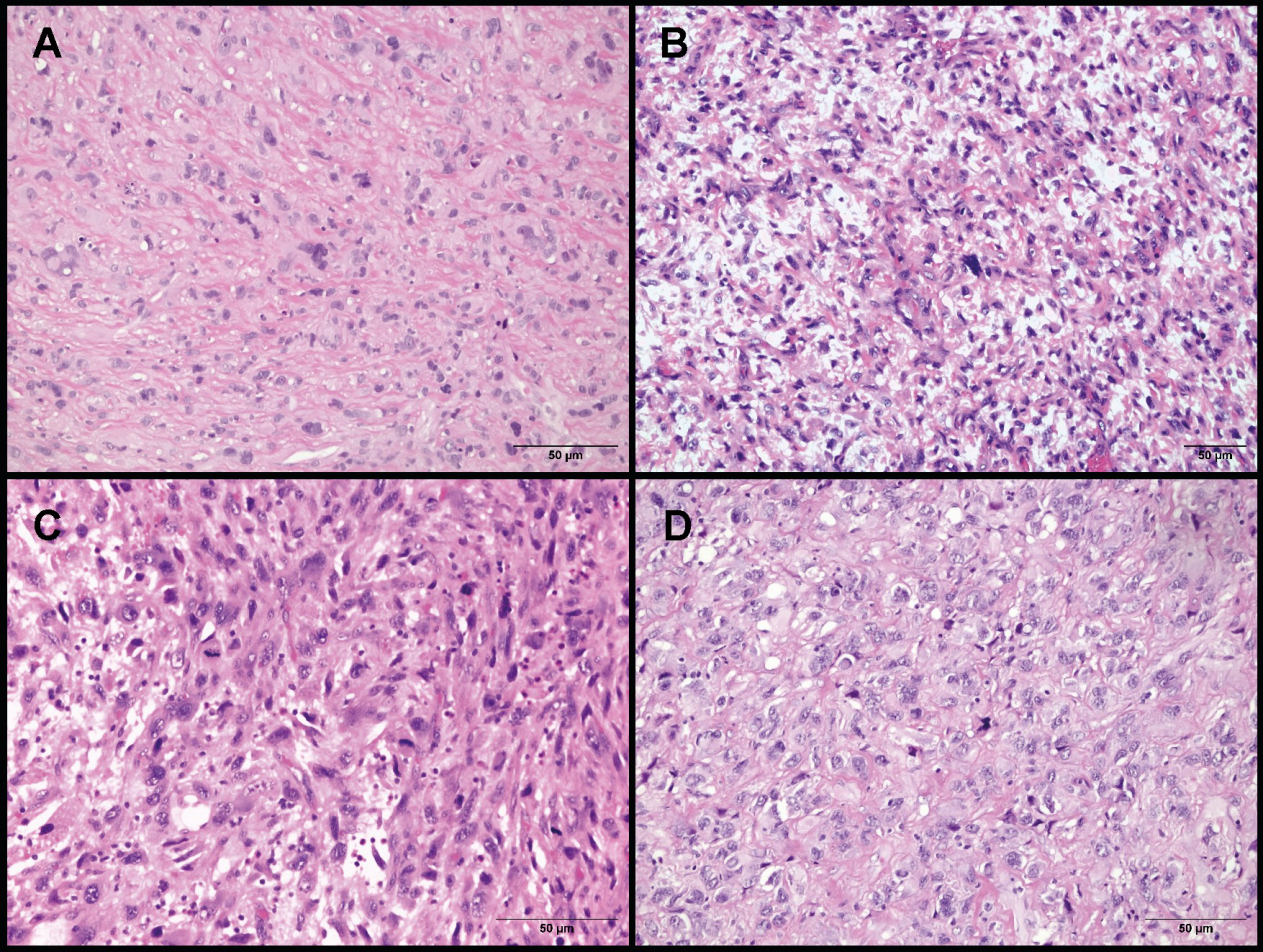
Histological characteristics of L328, L329, L331 and L332. **(A)** L328 presenting epithelioid cells, with irregularly shaped nuclei of variable size and weakly eosinophilic cytoplasm, embedded in a collagenous stroma. **(B)** L329 showing spindle and epithelioid cells mixed with pleomorphic elements. Numerous mitotic figures are present. **(C)** L331 with predominantly epithelioid and focally spindle cells associated with high mitotic activity. **(D)** L332 displaying epithelioid cells with pale cytoplasm and vesicular nuclei containing one or more prominent nucleoli, embedded in a collagenous stroma.

All samples presented dense proliferation of atypical cells often associated with multinucleated elements. Representative histologic images were available for only four cases as shown in **Figure 1**.

### 2.2 Whole exome and coding transcriptome sequencing

Formalin-fixed paraffin-embedded (FFPE) slides were reviewed by a pathologist and manually macrodissected to obtain an enrichment of tumor tissue of at least 70%. DNA was extracted from the tumor and coupled normal sample with the QIAamp DNA Micro Kit (Qiagen, Hilden, Germany). The Nextera DNA Flex kit (Illumina, San Diego, CA, United States) was adopted to synthesize whole exome libraries. Total RNA extraction was carried out for all the tumor samples using the RecoverAll Total Nucleic Acid Isolation Kit (Thermo Fisher Scientific, Waltham, MA, United States). Subsequently, cDNA libraries were synthesized from 100 ng of total RNA adopting the TruSeq RNA Exome kit (Illumina, San Diego, CA, United States). Whole exome sequencing (WES) and whole transcriptome sequencing (WTS) libraries were sized with Agilent DNA 7500 chips on the Bioanalyzer 2100 (Agilent Technologies, Taiwan) and quantified with a fluorometric assay (Quant-iT PicoGreen assay; Life Technologies, Carlsbad, CA, United States). Paired-end libraries were respectively sequenced at 100 and 80 bp on a NextSeq500 instrument (Illumina, San Diego, CA, United States). Lastly, the quality assessment of all FASTQ files was performed by FastQC and MultiQC (12, 13).

### 2.3 Copy number analysis

The intimal sarcoma copy number profile was defined. The alignment of trimmed WES reads on the reference human genome hg38 was carried out by BWA-mem, while sorting and indexing were performed by SAMtools (14, 15). GATK was adopted for duplicate marking, read group addition and base quality score recalibration (16). Recalibrated normal and tumor bam files were processed by EXCAVATOR2 (window size = 50,000) to detect copy number alterations (CNA), after determining the tumor purity with PUREE (17, 18). Since L328 did not present the normal counterpart, we compared the tumor sample to the normal counterparts of the other samples and selected only the events present in all the comparisons.

### 2.4 Immune cell profiling and mutation detection

Trimmed WTS reads were mapped on the reference human genome hg38 using STAR, followed by the removal of duplicate reads, indexing and sorting of the remaining reads by SAMtools (15, 19). Raw gene counts were obtained by the python package HTSeq-count and normalized as transcripts per million (TPM), to guarantee sample comparability (20, 21). To investigate the tumor microenvironment, CIBERSORTx was applied to the TPM matrix (22). This tool defines the absolute and relative abundance of immune cell types (LM22 signature in our case). Furthermore, the Tumor Inflammation Signature (TIS) score was calculated as the weighted sum of the log2-transformed TPM of the 18 TIS genes, normalized to 10 housekeeping genes, as previously described by Danaher et al. (23). Ultimately, gene fusions were identified from WTS FASTQ files using STAR-Fusion, while somatic variants were detected from recalibrated WES BAM files and annotated using Mutect2 and ANNOVAR, respectively (16, 24, 25). To calculate the tumor mutational burden (TMB) for the patients with matched tumor-normal samples, non-synonymous exonic and splicing variants with at least 3 reads supporting the alternate allele and the ratio between these reads and the total read number > 0.14 were included.

### 2.5 Differential gene expression analysis

To define the intimal sarcoma profile, we compared our samples with other sarcoma FFPE samples from the Gene Expression Omnibus (GEO) database (https://www.ncbi.nlm.nih.gov/geo/, accession number: GSE71120): 5 undifferentiated pleomorphic sarcomas (UPS) and 3 leiomyosarcomas (LMS) presenting an RNA quality score ≥ 3 (**Table 2**). UPS were introduced because of their undifferentiated state which characterizes intimal sarcomas as well, while LMS were included since they represent the most recurrent histotype of the vascular wall. Two leiomyosarcomas of the inferior vena cava analyzed in our lab were also added to the LMS subgroup (L538 and L539). An initial quality control of FASTQ files and batch correction with the sva package were performed, followed by TPM transformation as previously mentioned, to perform the principal component analysis (PCA) including the whole transcriptomic profile (26). The TPM matrix was also used for the cell type enrichment analysis by xCell and for the gene set enrichment analysis (GSEA) (IS *versus* UPS and IS *versus* LMS), selecting the Hallmark and Reactome datasets (27, 28). GSEA parameters were set as follows: “number of permutations” = “1000”, “permutation type” = “gene set”, “enrichment statistic” = “weighted”, “metric for ranking gene” = “Signal2Noise” and “normalization mode” = “meandiv”. Ultimately, to carry out the differential gene expression analysis (IS *versus* UPS and IS *versus* LMS), the R-bioconductor package edgeR was applied (29).

**Table 2:** Classification of the UPS and LMS included in the DGE analysis (GEO: GSE71120).

ID	Gender	Age	Histotype	Localization
SRR2065009	F	63	UPS^a^	trunk wall
SRR2065011	F	54	LMS^b^	internal trunk
SRR2065016	F	35	UPS^a^	lower limb
SRR2065017	F	82	UPS^a^	lower limb
SRR2065019	F	67	LMS^b^	GI tract^c^
SRR2065022	M	48	LMS^b^	lower limb
SRR2065024	M	63	UPS^a^	trunk wall
SRR2065106	F	24	UPS^a^	trunk wall
L538	F	NA^d^	LMS^b^	inferior vena cava
L539	F	69	LMS^b^	inferior vena cava

^a^UPS, Undifferentiated Pleomorphic Sarcoma; ^b^LMS, Leiomyosarcoma; ^c^GI tract, Gastrointestinal tract; ^d^Not Available.

## 3 Results

### 3.1 Copy number profiling: histotype-specific and novel marker amplification

**Figure 2:**
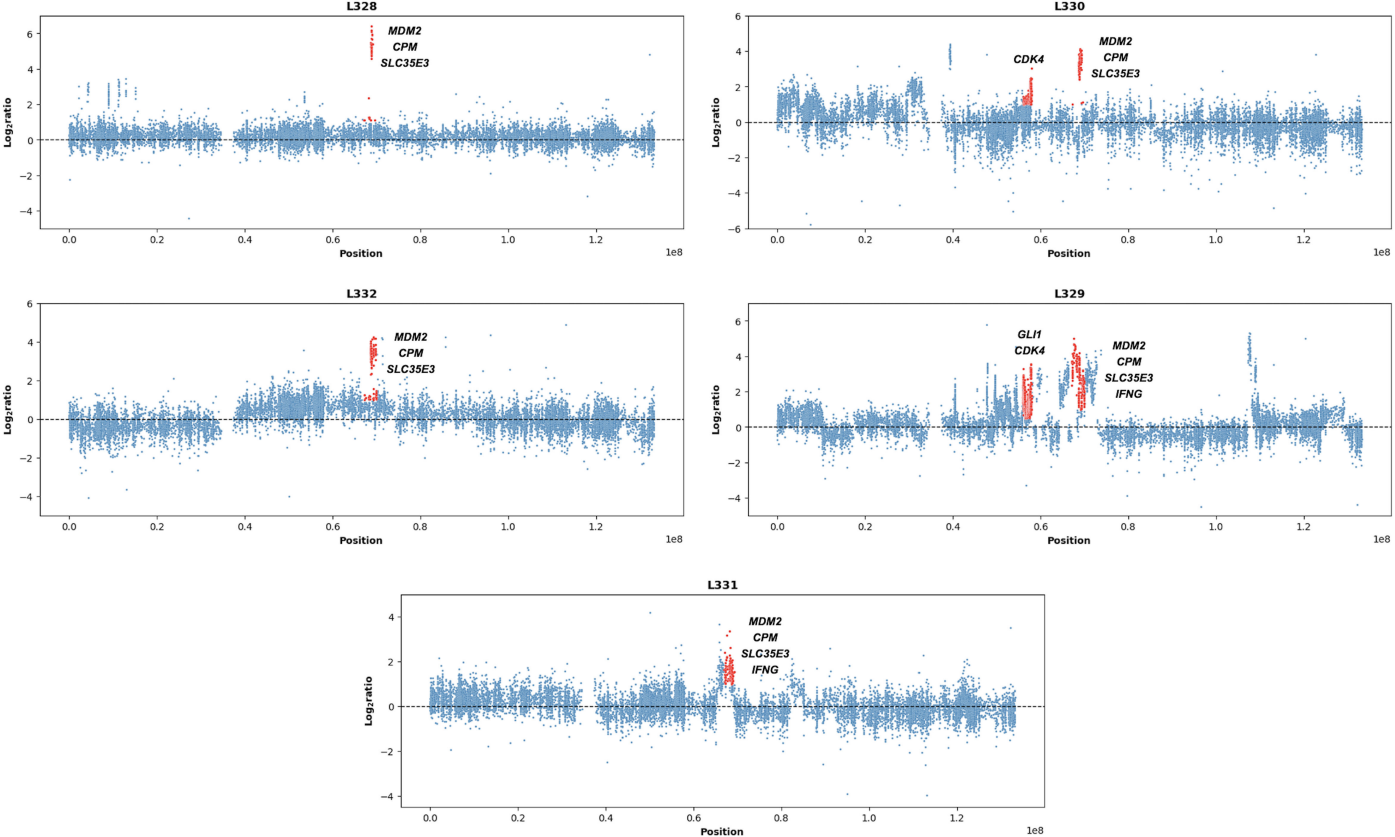
Chromosome 12 amplifications in our intimal sarcomas. Scatter plots representing chr12 copy number alterations in our cohort of intimal sarcomas (n=5). Relevant genes have been highlighted in red, especially those shared by all samples: *MDM2* (known marker), *CPM* and *SLC35E3* (novel markers). The x axis represents the genomic positions, while the y axis shows the log2ratio defined by EXCAVATOR2.

Copy number analysis was performed on 5 intimal sarcoma cases to confirm their diagnosis and detect events that might have negatively affected patients’ prognosis. As a result, they were all characterized by the *MDM2* amplification, which is the main biomarker of the histotype. This alteration was associated with amplifications of *CDK4* in L329 and L330, *IFNG* in L329 and L331, and *GLI1* in L329 on the same chromosome (**Figure 2**). Interestingly, both *CPM* and *SLC35E3* (12q15) were amplified in all samples (**Figure 2**). Other relevant events comprised *KIT* and *PDGFRA* (4q12) amplifications in L330 and L332, and *EGFR* (7p11.2) gain in L332. Conversely, no recurrent gene fusions or somatic mutations of significance were detected in our samples.

### 3.2 Immune cell population enrichment and PD-L1 expression

**Figure 3:**
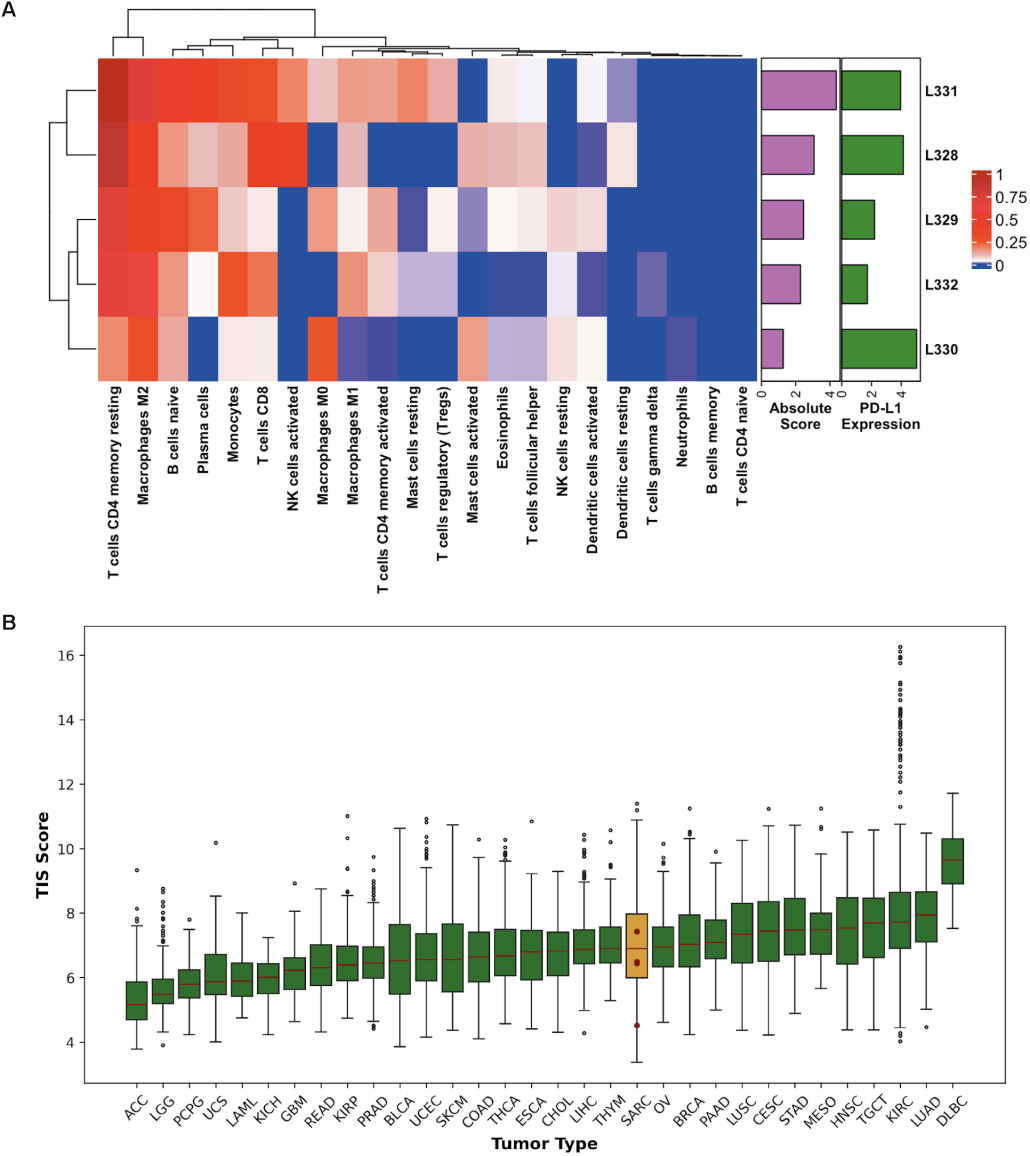
Integrated analysis of immune cell composition and TIS score. **(A)** Hierarchical clustering displaying the abundance of 22 immune cell types (columns) calculated by CIBERSORTx across our 5 intimal sarcomas (rows). Cell levels are displayed on a dark blue-to-dark red color scale. The two barplots represent the CIBERSORTx absolute score (violet) summarizing all the immune populations and the PD-L1 expression levels (green) in each sample. **(B)** Boxplot showing the TIS score (y axis) calculated for the TCGA groups (x axis) and our 5 intimal sarcomas, which are represented by the red dots: L331 = 7.43, L328 = 7.42, L332 = 6.49, L329 = 6.44 and L330 = 4.52. The median values correspond to the horizontal red lines, while the sarcoma subgroup is highlighted in dark yellow as it includes our cases. Outliers are displayed as empty dots. ACC, adrenocortical carcinoma; LGG, brain lower grade glioma; PCPG, pheochromocytoma and paraganglioma; UCS, uterine carcinosarcoma; LAML, acute myeloid leukemia; KICH, kidney chromophobe; GBM, glioblastoma multiforme; READ, rectum adenocarcinoma; KIRP, kidney renal papillary cell carcinoma; PRAD, prostate adenocarcinoma; BLCA, bladder urothelial carcinoma; UCEC, uterine corpus endometrial carcinoma; SKCM, skin cutaneous melanoma; COAD, colon adenocarcinoma; THCA, thyroid carcinoma; ESCA, esophageal carcinoma; CHOL, cholangiocarcinoma; LIHC, liver hepatocellular carcinoma; THYM, thymoma; SARC, sarcoma; OV, ovarian serous cystadenocarcinoma; BRCA, breast invasive carcinoma; PAAD, pancreatic adenocarcinoma; LUSC, lung squamous cell carcinoma; CESC, cervical squamous cell carcinoma and endocervical adenocarcinoma; STAD, stomach adenocarcinoma; MESO, mesothelioma; HNSC, head and neck squamous cell carcinoma; TGCT, testicular germ cell tumors; KIRC, kidney renal clear cell carcinoma; LUAD, lung adenocarcinoma; DLBC, diffuse large B-cell lymphoma.

The IS immunological profile was explored to evaluate immunotherapy as a promising therapeutic approach. The abundance of 22 immune cell types was quantified by CIBERSORTx, revealing a prevalence of CD4+ memory resting T-cells and M2 macrophages in all samples (*p*-value<0.050). The L328, L331 and L332 cases were also characterized by the presence of CD8+ T-cells and M1 macrophages, while L330 displayed the highest level of M0 macrophages. Naïve B-cells were detected in all samples at different concentrations (**Figure 3A**). Remarkably, L330 showed the lowest microenvironment score according to CIBERSORTx, while it presented the highest expression of PD-L1 (*CD274*), a PD1-ligand that can inhibit T-cell activation. Accordingly, the copy number analysis revealed *CD274* amplification in this sample. The same event was also detected in L331, which was characterized by the highest absolute score and tumor mutational burden (1.53 mut/Mb), even if the TMB was generally low across all the patients presenting the matched normal counterpart (L329 = 0.96 mut/Mb; L330 = 1.22 mut/Mb and L332 = 0.80 mut/Mb).

Moreover, the Tumor Inflammation Signature (TIS) score of our samples and other tumor subgroups from The Cancer Genome Atlas (TCGA) database was calculated. This score measures the downregulated adaptive immunity in tumor samples to establish their sensitivity to immune checkpoint inhibitors. Subsequently, we ordered all the TCGA histotypes according to their median TIS score (**Figure 3B**). Comparing our results to those of the sarcoma subset, except for the L330 outlier, our TIS scores are close to their median (6.90), with two of our values being above this threshold (L328 and L331).

### 3.3 Enhanced immune signaling in IS *versus* UPS and LMS

**Figure 4:**
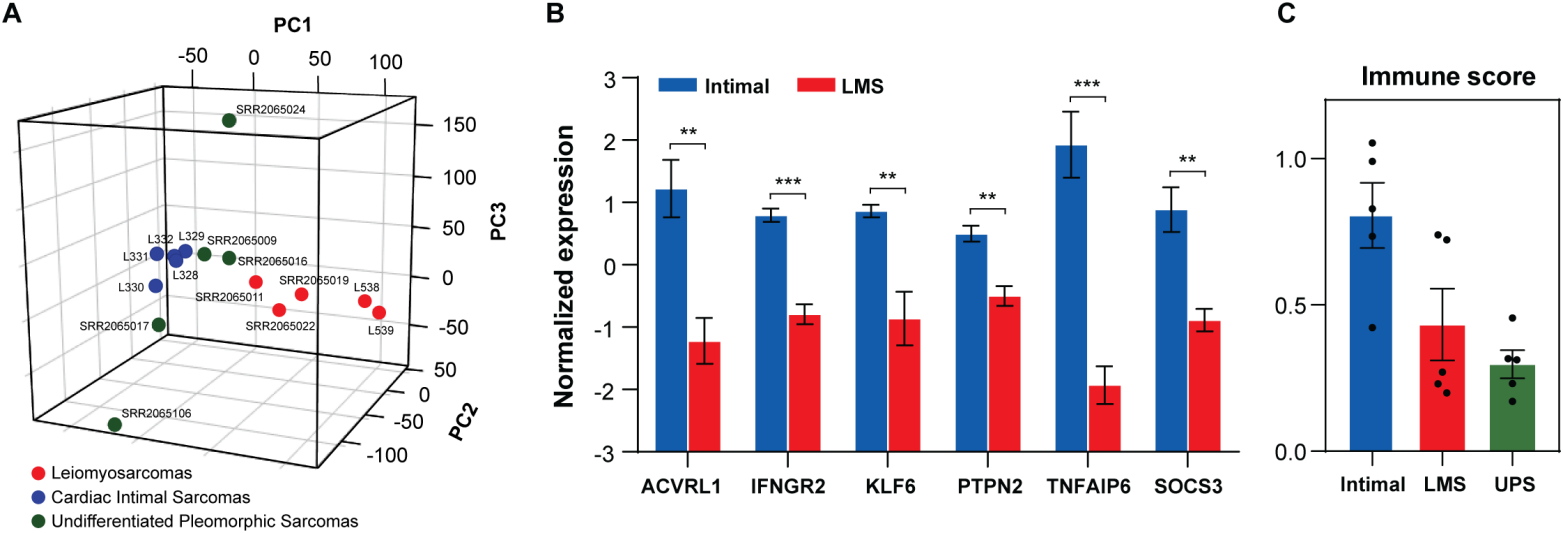
Molecular and immune characterization of our intimal sarcomas (n=5), leiomyosarcomas (n=5) and undifferentiated pleomorphic sarcomas (n=5). **(A)** 3D PCA plot (PC1 = 13.14%; PC2 = 12.88%; PC3 = 12.55%) presenting a cluster of our 5 intimal sarcomas (blue) surrounded by most undifferentiated pleomorphic sarcomas (green) and a more distant cluster of 5 leiomyosarcomas (red). **(B)** Barplot with the normalized expression (y axis) of genes (x axis) involved in immune-related pathways enriched in our 5 intimal sarcomas (blue) compared to 5 leiomyosarcomas (red) (unpaired t-test: **q-value<0.010; ***q-value<0.001). **(C)** The mean ± SE of the xCell immune score in intimal sarcomas (blue), leiomyosarcomas (red) and undifferentiated pleomorphic sarcomas (green). Black dots represent the samples.

The IS expression profile was compared to the one of 5 undifferentiated pleomorphic sarcomas (UPS), which also lack a clear histological differentiation profile, and to the gene signature of 5 leiomyosarcomas (LMS), which represent the most recurrent sarcoma histotype in the vascular wall. The unsupervised PCA showed a distinct cluster of intimal sarcomas surrounded by the UPS, supporting the undifferentiated profile they share (**Figure 4A**).

The DGE analysis also showed a close resemblance between IS and UPS with only 34 differentially expressed genes (*q*-value<0.050). Among the upregulated genes in IS, there are several encoding immunoglobulins (IGHV3-30, IGHV1-18, IGKV3-15, IGLV3–21 and IGHV3-15) and *NDE1* producing a protein for centrosome duplication and mitotic spindle formation. These findings complied with the enriched immune profile of our intimal sarcoma series. Conversely, IS presented more than 1000 differentially expressed genes when compared to the leiomyosarcomas. Intimal sarcomas showed low expression levels of the LMS differentiation markers (e.g. *ACTC1*, *MYOCD*, *ACTA1*) and downregulation of smooth muscle contraction (GSEA Reactome). More importantly, they were characterized by enriched pathways involved in the immune response compared to both LMS and UPS (GSEA Hallmark and Reactome) as shown in **Table 3**. The genes most contributing to the Hallmark pathways were significantly upregulated in the intimal sarcomas compared to the leiomyosarcomas (*q*-value<0.010 | *q*-value<0.001) (**Figure 4B**). Ultimately, the immune score calculated by xCell was higher in our intimal cases with respect to both LMS and UPS, with *p*-values of 0.054 and 0.003, respectively (**Figure 4C**). It is worth highlighting the influence of the high scores of L538 and L539, which are LMS of the inferior vena cava, on the former borderline *p*-value, suggesting a possible correlation between tumors arising from the vascular wall and their immune infiltrate levels.

**Table 3:** Most enriched immune-related pathways in intimal sarcomas *versus* LMS in red (GSEA Hallmark) and *versus* UPS in blue (GSEA Reactome).

Pathway	NES^a^	P-value	q-value
INFLAMMATORY RESPONSE	2.06	<0.001	<0.001
IL6/JAK/STAT3 SIGNALING	1.93	<0.001	0.001
TNF-α SIGNALING VIA NF-kB	1.79	<0.001	0.002
COMPLEMENT	1.62	<0.001	0.016
INTERFERON GAMMA RESPONSE	1.57	<0.001	0.023
TGF-β SIGNALING	1.53	0.012	0.028
mTORC1 SIGNALING	1.43	0.000	0.046
KRAS SIGNALING	1.43	0.006	0.050
CREATION OF C4 AND C2 ACTIVATORS	3.06	<0.001	<0.001
CD22 MEDIATED BCR REGULATION	3.02	<0.001	<0.001
INITIAL TRIGGERING OF COMPLEMENT	3.01	<0.001	<0.001
FCERI MEDIATED MAPK ACTIVATION	2.97	<0.001	<0.001
FCGR ACTIVATION	2.94	<0.001	<0.001
ANTIGEN ACTIVATES BCR	2.91	<0.001	<0.001
COMPLEMENT CASCADE	2.90	<0.001	<0.001
FCERI MEDIATED Ca^+2^ ACTIVATION	2.87	<0.001	<0.001

^a^NES, Normalized Enrichment Score.

## 4 Discussion

As of today, surgery remains the best treatment approach for localized intimal sarcomas and efficacious medical therapies are limited for unresectable and/or advanced tumors (30). Thus, despite the small sample size, we explored the IS genomic and immune profile attempting to uncover alternative therapeutic targets.

The copy number analysis confirmed the amplification of their known biomarker *MDM2* (mouse double minute 2 homolog) in all samples. A retrospective multicenter study showed that *MDM2*-positive patients with confined disease can partly benefit from anthracycline-based chemotherapy alone or in combination with other agents, with ~25% of patients expected to be disease-free after 2 years (5). Conversely, patients with advanced disease were characterized by a median progression-free survival of only 7.7 months (5). Thus, new treatment approaches such as targeted therapies should be implemented. The *MDM2*-inhibitor milademetan has been tested on patients with *MDM2*-amplified, *TP53* wild-type intimal sarcomas (31). Two of the enrolled patients (n=10) presented stable disease for > 15 months, while one case who withdrew from the study showed 32.7% tumor shrinkage. Nonetheless, other factors were brought into consideration, including *CDKN2A* copy-number loss and acquired *TP53* loss-of-function mutations which were negatively correlated with tumor response (31). Moreover, since *MDM2* was found co-amplified mostly with *CDK4*, *KIT* or *PDGFRA* in our samples, other inhibitors might be considered. The CDK4/6 kinases contribute to inhibiting the Rb tumor suppressor by phosphorylation. Consequently, CDK4/6 inhibitors such as palbociclib, ribociclib and abemaciclib have emerged (32). For instance, palbociclib has showed promising results in advanced well-differentiated or dedifferentiated liposarcomas, including *MDM2* downregulation (33). Tyrosine kinase inhibitors (TKI) have been particularly effective against gastrointestinal stromal tumors (GIST) with *KIT* and *PDGFRA* alterations. These genes encode type III tyrosine kinase receptors involved in relevant signaling cascades as the RAS–RAF–MAPK and PI3K–mTOR pathways (34, 35). Imatinib, sunitinib and regorafenib are, respectively, first-, second- and third-line TKI for GIST patients with *KIT* and most *PDGFRA* mutations (34, 35). Nevertheless, the synergistic effect of MDM2 and CDK4/TK inhibitors should be carefully evaluated due to potential interference and toxicity.

Remarkably, the concomitant amplification of both *CPM* and *SLC35E3* in all samples, likely related to their genomic proximity to *MDM2*, raised interest about their potential role as therapeutic targets and their impact on patient outcome. Little is known regarding their contribution to cancer development. However, the carboxypeptidase M (CPM) regulates peptide hormone and growth factor activity and has already been found co-amplified with *MDM2* in well-differentiated liposarcomas and with *EGFR* in lung adenocarcinomas, where it was associated with adverse prognosis (36, 37). It has also been demonstrated that inhibiting *CPM* downregulates *EGFR* activity, which contributes to cell growth, proliferation and survival (37). *SLC35E3* encodes a solute carrier family member and has been found co-amplified with *MDM2* in glioblastoma multiforme (GBM) cell lines (38). Thus, further investigation is warranted.

Alternatively, immunotherapy could be a viable option, especially considering PD-L1 expression in all our IS. Pembrolizumab, an immune checkpoint inhibitor targeting PD-1, has shown partial response in 3 cases of advanced *MDM2-*amplified intimal sarcomas with low tumor mutational burden and stable microsatellite (11). Since the tumor mass was reduced in all 3 patients, tumor response was likely influenced by the tumor microenvironment, PD-L1 expression and presence of tertiary lymphoid structures (11). Pembrolizumab was also associated with a positive objective response rate in undifferentiated pleomorphic sarcomas and dedifferentiated liposarcomas (39). Conversely, there have been rare cases of metastatic carcinoma treated with anti-PD-1/PD-L1 monotherapy where a positive correlation between hyper-progression and the presence of *MDM2* and *EGFR* alterations was observed (40). Since these events have been found in our cases as well, genetic alterations should be carefully considered prior to immunotherapy administration. Regarding the tumor microenvironment, a study described an active immune-rich milieu in 7 IS with a positive correlation between PD-L1 expression and the amount of CD45+, CD8+, FOXP3+, CD68+ and LAG3+ cells (10). Park et al. found an immune-inflamed phenotype prevalently in *MDM2*-wild-type IS (4/6 cases) but also in 2/8 *MDM2*-amplified samples, underlining IS heterogeneity (41). These findings suggest intimal sarcoma sensitivity to immunotherapy and are in line with our results, which showed a prevalence of CD4+ memory resting T-cells and macrophages M2, along with different concentrations of naïve B-cells, CD8+ T-cells and monocytes, in all our samples. Remarkably, L330 presented the lowest CIBERSORTx absolute, xCell immune and tumor inflammation signature (TIS) scores unlike L328 and L331, indicating a positive correlation among these scores. Moreover, since the TIS score represented a favorable prognostic factor in the TCGA sarcoma subgroup, patients with an enriched immune infiltrate are more likely to have a prolonged overall survival rate (23). L330, together with L331, also exhibited *CD274* amplification and high levels of PD-L1 expression, suggesting that constitutive, genomically driven PD-L1 expression may contribute in these tumors. Accordingly, the coexistence of adaptive and constitutive immune resistance represents a plausible explanation for the observed patterns and warrants further investigation.

Ultimately, the upregulation of immunoglobulins, pathways involved in the immune response (e.g. IL6/JAK/STAT3 and TNF-α via NF-kB signaling, interferon gamma response) and other markers (such as *NDE1, ACVRL1, KLF6, PTPN2, IFNGR2, TNFAIP6, SOCS3)* may serve as further indicators of amenability to immunotherapy. For instance, *NDE1* has been found highly expressed in several malignancies promoting cell proliferation and metastasis formation (42). It was recently found associated with poor prognosis, immune cell infiltration and expression of most immune checkpoint genes in several cancer types, suggesting a relationship between NDE1 levels and the responsiveness to checkpoint inhibitors (42). *ACVRL1* expression has caused resistance to tyrosine kinase inhibitors in colorectal cancer (43). Additionally, *ACVRL1* and *KLF6* have been associated with immune infiltration, while *PTPN2* loss seems to promote anti-tumor immune response (44–47). *IFNGR2* encodes one of the subunits of the IFN-γ receptor, whose dimerization leads to the JAK/STAT pathway activation. As previously mentioned, the gene encoding its ligand IFN-γ was also found amplified in two of our samples, which can enhance the receptor activation (48). *TNFAIP6* can lead to tumor progression and, consequently, poor prognosis but it might also promote the action of neutrophils (49–51). Lastly, due to its involvement in cancer progression, *SOCS3* knockdown has shown strong anti-cancer response in murine models (52, 53).

Our study presented some limitations mainly due to the rarity of the histotype, such as the small sample size of our case series. Moreover, restricting the analysis only to *MDM2*-amplified intimal sarcomas of the pulmonary artery may fail to capture the full heterogeneity of the histotype, potentially limiting the comprehensive view of the disease. Nevertheless, the pulmonary artery represents the most recurrent site of origin. It should also be acknowledged that, both in our work and in the existing literature, *in vitro* and *in vivo* experiments are lacking.

Despite these caveats, our research offered an overview of the main biomarkers and immune profile of 5 intimal sarcomas. The amplification of both histotype-specific (*MDM2*) and novel (*CPM* and *SLC35E3*) markers was identified in all samples, thus their potential role should be more defined in preclinical pharmacological studies. Additionally, all the cases were characterized by an enriched immune infiltrate, associated with the expression of PD-L1, immune-related genes and pathways. Therefore, these observations confirm and reinforce those published on the immune-inflamed phenotype in *MDM2*-amplified IS and underline the possibility to further explore alternative therapeutic strategy involving immunotherapy. In conclusion, these findings represent the preliminary basis for future immunological studies on IS including more cases, possibly from different sites to account for tumor heterogeneity.

## Data Availability

The datasets presented in this study can be found in online repositories. The names of the repository/repositories and accession number(s) can be found below: https://www.ncbi.nlm.nih.gov/, PRJNA896891 and PRJNA1333676.

## References

[1] Koelsche C Benhamida JK Kommoss FKF Stichel D Jones DTW Pfister SM . Intimal sarcomas and undifferentiated cardiac sarcomas carry mutually exclusive MDM2, MDM4, and CDK6 amplifications and share a common DNA methylation signature. Modern Pathol. (2021) 34:2122–9. doi: 10.1038/s41379-021-00874-y, PMID: 34312479 PMC8592836

[2] Giner F MaChado I Rubio-Martínez LA López-Guerrero JA Claramunt-Alonso R Navarro S . Intimal sarcoma with MDM2/CDK4 amplification and p16 overexpression: A review of histological features in primary tumor and xenograft, with immunophenotype and molecular profiling. Int J Mol Sci. (2023) 24:7535. doi: 10.3390/ijms24087535, PMID: 37108696 PMC10141691

[3] Alizadehasl A Najdaghi S Mohseni Salehi M Meshgi S Hosseini Jebelli SF Yalameh Aliabadi A . A comprehensive insight into primary intimal sarcoma of the pulmonary artery; from diagnosis to management: A case report and review of the literature. Clin Case Rep. (2024) 12:e9580. doi: 10.1002/ccr3.9580, PMID: 39563855 PMC11573721

[4] Bode-Lesniewska B Zhao J Speel EJM Biraima AM Turina M Komminoth P . Gains of 12q13–14 and overexpression of mdm2 are frequent findings in intimal sarcomas of the pulmonary artery. Virchows Archiv. (2001) 438:57–65. doi: 10.1007/s004280000313, PMID: 11213836

[5] Frezza AM Assi T Lo Vullo S Ben-Ami E Dufresne A Yonemori K . Systemic treatments in MDM2 positive intimal sarcoma: A multicentre experience with anthracycline, gemcitabine, and pazopanib within the World Sarcoma Network. Cancer. (2020) 126:98–104. doi: 10.1002/cncr.32508, PMID: 31536651 PMC9187112

[6] WHO . Classification of Tumours Editorial Board. Soft tissue and bone tumours Vol. 3. . Lyon (France: International Agency for Research on Cancer (2020).

[7] Oliner JD Saiki AY Caenepeel S . The role of MDM2 amplification and overexpression in tumorigenesis. Cold Spring Harb Perspect Med. (2016) 6:a026336. doi: 10.1101/cshperspect.a026336, PMID: 27194168 PMC4888815

[8] Koo N Sharma AK Narayan S . Therapeutics targeting p53-MDM2 interaction to induce cancer cell death. Int J Mol Sci. (2022) 23:5005. doi: 10.3390/ijms23095005, PMID: 35563397 PMC9103871

[9] Van Dievel J Sciot R Delcroix M Vandeweyer R Debiec-Rychter M Dewaele B . Single-center experience with intimal sarcoma, an ultra-orphan, commonly fatal mesenchymal Malignancy. J Clin Oncol. (2017) 35:e22532–2. doi: 10.1159/000476036, PMID: 28501860

[10] Birkness-Gartman JE Thomas DL Engle LL Voltaggio L Thompson ED . Immune microenvironment of intimal sarcomas: Adaptive immune resistance with potential therapeutic implications. Am J Clin Pathol. (2024) 161:256–63. doi: 10.1093/ajcp/aqad14210, PMID: 37921094

[11] Ribeiro MF Demicco EG Razak ARA . Clinical activity of pembrolizumab in refractory MDM2-amplified advanced intimal sarcomas. Ther Adv Med Oncol. (2024) 16. doi: 10.1177/17588359241250158, PMID: 38745586 PMC11092541

[12] Andrew S . 2010. FastQC: A quality control tool for high throughput sequence data. Available online at: http://www.bioinformatics.babraham.ac.uk/projects/fastqc/ (Accessed November 14, 2024).

[13] Ewels P Magnusson M Lundin S Käller M . MultiQC: summarize analysis results for multiple tools and samples in a single report. Bioinformatics. (2016) 32:3047–8. doi: 10.1093/bioinformatics/btw354, PMID: 27312411 PMC5039924

[14] Li H . Aligning sequence reads, clone sequences and assembly contigs with BWA-MEM. (2013). doi: 10.48550/arXiv.1303.3997, PMID: 41363103

[15] Danecek P Bonfield JK Liddle J Marshall J Ohan V Pollard MO . Twelve years of SAMtools and BCFtools. Gigascience. 2021:giab008. doi: 10.1093/gigascience/giab008, PMID: 33590861 PMC7931819

[16] Van der Auwera GA Carneiro MO Hartl C Poplin R del Angel G Levy-Moonshine A . From fastQ data to high-confidence variant calls: the genome analysis toolkit best practices pipeline. Curr Protoc Bioinf. (2013) 43:11.10.1–11.10.33. doi: 10.1002/0471250953.bi1110s43, PMID: 25431634 PMC4243306

[17] D’Aurizio R Pippucci T Tattini L Giusti B Pellegrini M Magi A . Enhanced copy number variants detection from whole-exome sequencing data using EXCAVATOR2. Nucleic Acids Res. (2016) 44:e154. doi: 10.1093/nar/gkw695, PMID: 27507884 PMC5175347

[18] Revkov E Kulshrestha T Sung KWK Skanderup AJ . PUREE: accurate pan-cancer tumor purity estimation from gene expression data. Commun Biol. (2023) 6:394. doi: 10.1038/s42003-023-04764-8, PMID: 37041233 PMC10090153

[19] Dobin A Davis CA Schlesinger F Drenkow J Zaleski C Jha S . STAR: ultrafast universal RNA-seq aligner. Bioinformatics. (2013) 29:15–21. doi: 10.1093/bioinformatics/bts635, PMID: 23104886 PMC3530905

[20] Anders S Pyl PT Huber W . HTSeq--a Python framework to work with high-throughput sequencing data. Bioinformatics. (2015) 31:166–9. doi: 10.1093/bioinformatics/btu638, PMID: 25260700 PMC4287950

[21] R Core Team . R: A language and environment for statistical computing. R Foundation for Statistical Computing, Vienna (2023). Available online at: https://www.R-project.org/ (Accessed: December 10, 2024).

[22] Newman AM Steen CB Liu CL Gentles AJ Chaudhuri AA Scherer F . Determining cell type abundance and expression from bulk tissues with digital cytometry. Nat Biotechnol. (2019) 37:773–82. doi: 10.1038/s41587-019-0114-2, PMID: 31061481 PMC6610714

[23] Danaher P Warren S Lu R Samayoa J Sullivan A Pekker I . Pan-cancer adaptive immune resistance as defined by the Tumor Inflammation Signature (TIS): results from The Cancer Genome Atlas (TCGA). J Immunother Cancer. (2018) 6:63. doi: 10.1186/s40425-018-0367-1, PMID: 29929551 PMC6013904

[24] Haas BJ Dobin A Stransky N Li B Yang X Tickle T . STAR-fusion: fast and accurate fusion transcript detection from RNA-seq. bioRxiv. (2017). doi: 10.1101/120295

[25] Wang K Li M Hakonarson H . ANNOVAR: functional annotation of genetic variants from high-throughput sequencing data. Nucleic Acids Res. (2010) 38:e164–4. doi: 10.1093/nar/gkq603, PMID: 20601685 PMC2938201

[26] Leek J Johnson W Parker H Fertig E Jaffe A Zhang Y . sva: surrogate variable analysis. (2025). doi: 10.18129/B9.bioc.sva, PMID: 41206936

[27] Aran D Hu Z Butte AJ . xCell: digitally portraying the tissue cellular heterogeneity landscape. Genome Biol. (2017) 18:220. doi: 10.1186/s13059-017-1349-1, PMID: 29141660 PMC5688663

[28] Subramanian A Tamayo P Mootha VK Mukherjee S Ebert BL Gillette MA . Gene set enrichment analysis: A knowledge-based approach for interpreting genome-wide expression profiles. Proc Natl Acad Sci. (2005) 102:15545–50. doi: 10.1073/pnas.0506580102, PMID: 16199517 PMC1239896

[29] Robinson MD McCarthy DJ Smyth GK . edgeR: a Bioconductor package for differential expression analysis of digital gene expression data. Bioinformatics. (2010) 26:139–40. doi: 10.1093/bioinformatics/btp616, PMID: 19910308 PMC2796818

[30] Gronchi A Miah AB Dei Tos AP Abecassis N Bajpai J Bauer S . Soft tissue and visceral sarcomas: ESMO–EURACAN–GENTURIS Clinical Practice Guidelines for diagnosis, treatment and follow-up☆. Ann Oncol. (2021) 32:1348–65. doi: 10.1016/j.annonc.2021.07.006, PMID: 34303806

[31] Koyama T Shimizu T Kojima Y Sudo K Okuma HS Shimoi T . Clinical activity and exploratory resistance mechanism of milademetan, an MDM2 inhibitor, in intimal sarcoma with *MDM2* amplification: an open-label phase ib/II study. Cancer Discov. (2023) 13:1814–25. doi: 10.1158/2159-8290.cd-23-0419, PMID: 37369013

[32] Merlini A Pavese V Manessi G Rabino M Tolomeo F Aliberti S . Targeting cyclin-dependent kinases in sarcoma treatment: Current perspectives and future directions. Front Oncol. (2023) 13. doi: 10.3389/fonc.2023.1095219, PMID: 36741019 PMC9893281

[33] Dickson MA Schwartz GK Keohan ML D’Angelo SP Gounder MM Chi P . Progression-free survival among patients with well-differentiated or dedifferentiated liposarcoma treated with CDK4 inhibitor palbociclib. JAMA Oncol. (2016) 2:937. doi: 10.1001/jamaoncol.2016.0264, PMID: 27124835 PMC4991028

[34] Blay JY Kang YK Nishida T von Mehren M . Gastrointestinal stromal tumours. Vol. 7, Nature Reviews Disease Primers. Nat Res. (2021) 7:22. doi: 10.1038/s41572-021-00254-5, PMID: 33737510

[35] Bauer S George S von Mehren M Heinrich MC . Early and next-generation KIT/PDGFRA kinase inhibitors and the future of treatment for advanced gastrointestinal stromal tumor. Front Oncol. (2021) 11. doi: 10.3389/fonc.2021.672500, PMID: 34322383 PMC8313277

[36] Denis CJ Deiteren K Hendriks D Proost P Lambeir AM . Carboxypeptidase M in apoptosis, adipogenesis and cancer. Clin Chimi Acta. (2013) 415:306–16. doi: 10.1016/j.cca.2012.11.012, PMID: 23178445

[37] Kanojia D Nagata Y Garg M Lee DH Sato A Yoshida K . Genomic landscape of liposarcoma. Oncotarget. (2015) 6:42429–44. doi: 10.18632/oncotarget.6464, PMID: 26643872 PMC4767443

[38] Hodgson JG Yeh RF Ray A Wang NJ Smirnov I Yu M . Comparative analyses of gene copy number and mRNA expression in glioblastoma multiforme tumors and xenografts. Neuro Oncol. (2009) 11:477–87. doi: 10.1215/15228517-2008-113, PMID: 19139420 PMC2765338

[39] Tawbi HA Burgess M Bolejack V Van Tine BA Schuetze SM Hu J . Pembrolizumab in advanced soft-tissue sarcoma and bone sarcoma (SARC028): a multicentre, two-cohort, single-arm, open-label, phase 2 trial. Lancet Oncol. (2017) 18:1493–501. doi: 10.1016/s1470-2045(17)30624-1, PMID: 28988646 PMC7939029

[40] Kato S Goodman A Walavalkar V Barkauskas DA Sharabi A Kurzrock R . Hyperprogressors after immunotherapy: analysis of genomic alterations associated with accelerated growth rate. Clin Cancer Res. (2017) 23:4242–50. doi: 10.1158/1078-0432.ccr-16-3133, PMID: 28351930 PMC5647162

[41] Park C Kim R Bae JM Lee T Song S Kwak Y . Genomic profiling of intimal sarcoma reveals molecular subtypes with distinct tumor microenvironments and therapeutic implications. ESMO Open. (2025) 10:104097. doi: 10.1016/j.esmoop.2024.104097, PMID: 39778225 PMC11758979

[42] Wang P Ning J Chen W Zou F Yu W Rao T . Comprehensive analysis indicated that NDE1 is a potential biomarker for pan-cancer and promotes bladder cancer progression. Cancer Med. (2024) 13:e6931. doi: 10.1002/cam4.6931, PMID: 38466053 PMC10926885

[43] Lu X Liu R Liao Y Cui L Sun H Zhang D . ACVRL1 drives resistance to multitarget tyrosine kinase inhibitors in colorectal cancer by promoting USP15-mediated GPX2 stabilization. BMC Med. (2023) 21:366. doi: 10.1186/s12916-023-03066-4, PMID: 37743483 PMC10518977

[44] Bocci M Sjölund J Kurzejamska E Lindgren D Marzouka NAD Bartoschek M . Activin receptor-like kinase 1 is associated with immune cell infiltration and regulates CLEC14A transcription in cancer. Angiogenesis. (2019) 22:117–31. doi: 10.1007/s10456-018-9642-5, PMID: 30132150 PMC6510886

[45] Lin J Liu P Sun K Jiang L Liu Y Huang Y . Comprehensive analysis of KLF family reveals KLF6 as a promising prognostic and immune biomarker in pancreatic ductal adenocarcinoma. Cancer Cell Int. (2024) 24:177. doi: 10.1186/s12935-024-03369-3, PMID: 38773440 PMC11106939

[46] Manguso RT Pope HW Zimmer MD Brown FD Yates KB Miller BC . *In vivo* CRISPR screening identifies Ptpn2 as a cancer immunotherapy target. Nature. (2017) 547:413–8. doi: 10.1038/nature23270, PMID: 28723893 PMC5924693

[47] Wiede F Lu K Du X Liang S Hochheiser K Dodd GT . PTPN 2 phosphatase deletion in T cells promotes anti-tumour immunity and CAR T-cell efficacy in solid tumours. EMBO J. (2020) 39:e103637. doi: 10.15252/embj.2019103637, PMID: 31803974 PMC6960448

[48] Fenton SE Saleiro D Platanias LC . Type I and II interferons in the anti-tumor immune response. Cancers (Basel). (2021) 13:1037. doi: 10.3390/cancers13051037, PMID: 33801234 PMC7957896

[49] Chan TC Li CF Ke HL Wei YC Shiue YL Li CC . High TNFAIP6 level is associated with poor prognosis of urothelial carcinomas. Urol Oncol Semin Orig Investig. (2019) 37:293.e11–293.e24. doi: 10.1016/j.urolonc.2018.12.009, PMID: 30595463

[50] Zhang X Xue J Yang H Zhou T Zu G . TNFAIP6 promotes invasion and metastasis of gastric cancer and indicates poor prognosis of patients. Tissue Cell. (2021) 68:101455. doi: 10.1016/j.tice.2020.101455, PMID: 33221562

[51] Liu R Zhu G Sun Y Li M Hu Z Cao P . Neutrophil infiltration associated genes on the prognosis and tumor immune microenvironment of lung adenocarcinoma. Front Immunol. (2023) 14. doi: 10.3389/fimmu.2023.1304529, PMID: 38204755 PMC10777728

[52] Dai L Tao Y Shi Z Liang W Hu W Xing Z . SOCS3 acts as an onco-immunological biomarker with value in assessing the tumor microenvironment, pathological staging, histological subtypes, therapeutic effect, and prognoses of several types of cancer. Front Oncol. (2022) 12. doi: 10.3389/fonc.2022.881801, PMID: 35600392 PMC9122507

[53] Mise-Omata S Ando M Srirat T Nakagawara K Hayakawa T Iizuka-Koga M . SOCS3 deletion in effector T cells confers an anti-tumorigenic role of IL-6 to the pro-tumorigenic cytokine. Cell Rep. (2023) 42:112940. doi: 10.1016/j.celrep.2023.112940, PMID: 37582370

